# Editorial: The Crossroads Between Immunological Disorders and Neuropsychiatric Diseases. A Case for Schizophrenia

**DOI:** 10.3389/fncel.2021.733997

**Published:** 2021-08-05

**Authors:** Silvia Sánchez-Ramón, Florence Faure, Stephen Jolles, Marion Leboyer, Marie-Ève Tremblay

**Affiliations:** ^1^Department of Clinical Immunology, Instituto de Medicina de Laboratorio and Instituto de Investigación Sanitaria San Carlos, Hospital Clínico San Carlos, Madrid, Spain; ^2^INSERM U932, PSL Research University, Institut Curie, Paris, France; ^3^Immunodeficiency Centre for Wales, University Hospital of Wales, Cardiff, United Kingdom; ^4^Université Paris Est Créteil, INSERM, IMRB, Translational Neuropsychiatry, Créteil, France; ^5^AP-HP, Hôpitaux Universitaires H. Mondor, DMU IMPACT, FHU ADAPT, Créteil, France; ^6^Division of Medical Sciences, University of Victoria, Victoria, BC, Canada; ^7^Axe Neurosciences, Centre de Recherche du Centre Hospitalier de l'Université de Montréal de Québec-Université Laval, Québec, QC, Canada; ^8^Department of Molecular Medicine, Faculty of Medicine, Université Laval, Québec, QC, Canada; ^9^Neurology and Neurosurgery Department, McGill University, Montréal, QC, Canada; ^10^Department of Biochemistry and Molecular Biology, The University of British Columbia, Vancouver, BC, Canada

**Keywords:** schizophrenia, maternal immune activation, innate immune system, self, neuroinflammation

Schizophrenia (SZ) is a prevalent psychiatric illness with a strong and complex genetic basis as well as wide spectrum of clinical manifestations (Gejman et al., 2010). Environmental inflammatory insults early in life and altered social behaviors have long been recognized to trigger the development of the disease (Zubin and Spring, 1977; Müller, 2018). In this Research Topic, we have compiled complementary articles from scientists working across diverse disciplines: original research studies conducted in human and experimental models, mini-reviews and reviews, as well as hypothesis and opinion articles aimed at clarifying the contribution of neuroimmunological interactions and inflammation to SZ.

In mouse models, Mi et al. explore new concepts of SZ as an acquired channelopathy. They suggest that specific ion channel alterations (voltage-gated sodium and small conductance calcium-activated potassium) induced by maternal immune activation (MIA)—by poly I:C challenge—combined to social isolation may in part account for the altered intrinsic neuronal excitability properties observed within the prefrontal cortex. The role of these channels in SZ-like behaviors warrants further examination. In another poly I:C-induced MIA mouse model of SZ, Ibi et al. focus on the implication of reelin—an extracellular matrix protein involved in neural development and synaptic plasticity—in the cognitive and emotional deficits in SZ. They investigate the potential role of reelin as a new molecular target for the treatment of neurodevelopmental disorders beyond the classical monoamine medication. In human studies, Hughes et al. further address the innate immune dysfunction in a cohort of 25 first episode psychosis patients compared to healthy controls. First episode psychosis patients were further grouped based on their presence or absence of mood disorder. The results suggest that dysfunctional monocyte responses are present in both affective and non-affective psychotic disorder, with a higher proinflammatory profile observed in monocytes/macrophages from patients with an affective psychotic disorder. In the context of the current COVID-19 pandemics, exploration of the potential relevance for future work studying outcomes in children of mothers who acquired COVID-19 during pregnancy as a putative mechanism for MIA will be of great interest to the scientific community.

Bridging the complex interplay of genetic and environmental etiologies described in SZ, the immune response and inflammatory events offer plausible explanations for the wide spectrum of disease symptoms. Choudhury and Lennox provide a comprehensive review on the epidemiological, genome wide association studies (GWAS), and animal models linking MIA and the complement system activation during specific gestational timings with the increased susceptibility to SZ in exposed offspring. Again very timely study, given the complement activation described in COVID-19 and the potential occurrence of infection during pregnancy (Java et al., 2020). Comer et al. discuss how genetic and environmental risk factors for SZ converge to alter microglial function during development, adolescence and adulthood, in response to systemic and central inflammation; as well as the role of the gut-brain axis. They introduce the microglial sensome as a group of receptors and ligands that enable microglia to sense and react to their changing environment and to modulate multiple cell-types in the brain to push vulnerable individuals above a certain threshold into a disease state (Comer et al.). In this framework, Bordeleau et al. provide a critical overview on the effects of maternal diet on the body's regulatory supersystems of the mother, such as the immune, endocrine, and nervous systems. The authors argue that altered gut microbiome and inadequate nutrient intake can not only affect maternal mental and physical health, but also induce a malabsorptive and inflammatory status that may predispose to neurodevelopmental disorders including SZ. Reale et al. review the key role of cytokines as mediators of immune activation and how imbalances of T helper cell (Th)1/Th2/Th17/regulatory T cell (Treg) influence the dopaminergic, noradrenergic, and serotonergic neurotransmission. They also discuss promising therapeutic strategies aimed at targeting cytokines/cytokine receptors in neurological disorders. A particularly original insight presenting SZ as an inflammatory-triggered neurovascular developmental disease is next given by Carrier et al.. In this mini-review, the potential alterations of the brain vasculature network in early postnatal life in specific regions contributing to the development of SZ are discussed, with key roles of some molecules in the SZ vascular signature, such as defects in claudin-5 and vascular endothelial growth factor signaling. The authors dissect the role played by the different neurovascular unit elements, such as pericytes, endothelial cells, astrocytes, and microglia, to maintain appropriate brain function and behavior.

Two intriguing hypotheses are further presented. Lucchese et al. elucidate the associations between human cytomegalovirus (HCMV) infection, aberrant neuronal migration, and psychosis, building on previous research that had assessed peptide commonality and potential immune cross-reactivity between microbial and human proteins. Numerous human proteins related to neuronal migration are involved in a specific heptapeptide overlap with HCMV. Sánchez-Ramón and Faure also conceptualize SZ as an autophrenic disease, in which autoreactive neurons and engrams interfere with normal discrimination of internal and external signals. They build on an analogy with the immune system based on self-instruction and recognition in the thymus gland to propose specific self-recognition at the cortex subplate during development. Lastly, Maes and Anderson close this Research topic with a thought-provoking opinion article on the “false conceptualizations” of SZ in the pursuit of a holistic view of the disorder and propose a new nomothetic network-model framework based on machine learning methods to track “evidence based” progress through model group-based classification across stages.

## Author Contributions

All authors listed have made a substantial, direct and intellectual contribution to the work, and approved it for publication.

## Conflict of Interest

The authors declare that the research was conducted in the absence of any commercial or financial relationships that could be construed as a potential conflict of interest.

## Publisher's Note

All claims expressed in this article are solely those of the authors and do not necessarily represent those of their affiliated organizations, or those of the publisher, the editors and the reviewers. Any product that may be evaluated in this article, or claim that may be made by its manufacturer, is not guaranteed or endorsed by the publisher.
